# Antibiotic resistance, bacterial transmission and improved prediction of bacterial infection in patients with antibody deficiency

**DOI:** 10.1093/jacamr/dlad135

**Published:** 2023-12-14

**Authors:** Sylvia Rofael, Clara Leboreiro Babe, Mehmet Davrandi, Alexandra L Kondratiuk, Leanne Cleaver, Naseem Ahmed, Claire Atkinson, Timothy McHugh, David M Lowe

**Affiliations:** Centre for Clinical Microbiology, University College London, Royal Free Campus, Pond Street, London, UK; Faculty of Pharmacy, University of Alexandria, Alexandria, Egypt; Centre for Clinical Microbiology, University College London, Royal Free Campus, Pond Street, London, UK; Centre for Clinical Microbiology, University College London, Royal Free Campus, Pond Street, London, UK; Institute for Immunity and Transplantation, Division of Infection and Immunity, University College London, Pears Building, Rowland Hill Street, London, UK; Centre for Host-Microbiome Interactions, Faculty of Dentistry, Oral & Craniofacial Sciences, Guy’s Campus, King’s College London, London, UK; Centre for Clinical Microbiology, University College London, Royal Free Campus, Pond Street, London, UK; Institute for Immunity and Transplantation, Division of Infection and Immunity, University College London, Pears Building, Rowland Hill Street, London, UK; Cancer Biology and Therapy Research Group, Divisionof Human Sciences, School of Applied Sciences, London South Bank University, London, UK; Centre for Clinical Microbiology, University College London, Royal Free Campus, Pond Street, London, UK; Institute for Immunity and Transplantation, Division of Infection and Immunity, University College London, Pears Building, Rowland Hill Street, London, UK; Department of Clinical Immunology, Royal Free London NHS Foundation Trust, Pond Street, London, UK

## Abstract

**Background:**

Antibody-deficient patients are at high risk of respiratory tract infections. Many therefore receive antibiotic prophylaxis and have access to antibiotics for self-administration in the event of breakthrough infections, which may increase antimicrobial resistance (AMR).

**Objectives:**

To understand AMR in the respiratory tract of patients with antibody deficiency.

**Methods:**

Sputum samples were collected from antibody-deficient patients in a cross-sectional and prospective study; bacteriology culture, 16S rRNA profiling and PCR detecting macrolide resistance genes were performed. Bacterial isolates were identified using MALDI-TOF, antimicrobial susceptibility was determined by disc diffusion and WGS of selected isolates was done using Illumina NextSeq with analysis for resistome and potential cross-transmission. Neutrophil elastase was measured by a ProteaseTag immunoassay.

**Results:**

Three hundred and forty-three bacterial isolates from sputum of 43 patients were tested. Macrolide and tetracycline resistance were common (82% and 35% of isolates). *erm*(B) and *mef*(A) were the most frequent determinants of macrolide resistance. WGS revealed viridans streptococci as the source of AMR genes, of which 23% also carried conjugative plasmids linked with AMR genes and other mobile genetic elements. Phylogenetic analysis of *Haemophilus influenzae* isolates suggested possible transmission between patients attending clinic.

In the prospective study, a negative correlation between sputum neutrophil elastase concentration and Shannon entropy α-diversity (Spearman’s ρ = −0.306, *P* = 0.005) and a positive relationship with Berger–Parker dominance index (ρ = 0.502, *P* < 0.001) were found. Similar relationships were noted for the change in elastase concentration between consecutive samples, increases in elastase associating with reduced α-diversity.

**Conclusions:**

Measures to limit antibiotic usage and spread of AMR should be implemented in immunodeficiency clinics. Sputum neutrophil elastase may be a useful marker to guide use of antibiotics for respiratory infection.

## Introduction

Microbial antibiotic resistance is one of the most urgent issues threatening provision of medical care.^1,2^ Underlying factors include the widespread use of antibiotics together with transmission of resistant organisms between vulnerable patients in clinical settings.^2,3^

Antibody-deficient patients, many of whom have bronchiectasis or other underlying lung disease, remain vulnerable to respiratory tract infection despite immunoglobulin replacement therapy.^4^ In a previous study, we observed frequent respiratory exacerbations in patients with common variable immunodeficiency (CVID), which were often self-treated with antibiotics.^5^ However, many infections in these patients are likely to have a viral aetiology.^5,6^ We noted that increasing sputum purulence, suggestive of neutrophilic inflammation, was a strong indicator of rapid response to antibiotics.^5^ Similarly, others have reported that neutrophil elastase increases significantly at bacterial exacerbation in patients with bronchiectasis.^7^ We have also reported a significantly abnormal respiratory microbiome in this patient population with markedly reduced α-diversity, which correlates negatively with neutrophil elastase or matrix metalloproteinase-9, both enzymes implicated in destruction of lung parenchyma and development of bronchiectasis.^8^ α-Diversity metrics indicate the richness and/or evenness of microbial populations, with decreases implying greater dominance of individual (often pathogenic) taxa. While it is important to treat bacterial pathogens and to prevent lung damage, excessive or poorly targeted use of antibiotics risks driving antimicrobial resistance.

In addition to frequent antibiotic treatment courses (0.36 courses per patient month in our previous study^5^), many antibody-deficient patients receive prophylactic antibiotics of various classes.^9^ Prophylactic azithromycin reduces the incidence of breakthrough respiratory tract infection but does not affect lung function and may be associated with high rates of resistance.^9^

It has previously been hypothesized that transmission of pathogenic organisms (specifically *Mycoplasma amphoriforme*) occurs between immunodeficient patients within clinical services,^10^ and this may include antibiotic-resistant bacteria.

We therefore investigated phenotypic and molecular antibiotic resistance within a cohort of antibody-deficient patients, sought to establish possible transmission of genetic elements encoding antibiotic resistance and to test whether changes in sputum neutrophil elastase level associate with bacterial infection.

## Materials and methods

### Patient cohorts and samples

Patients were eligible if they had a diagnosis of primary or secondary antibody deficiency requiring immunoglobulin replacement therapy and could spontaneously produce sputum. We recruited the first cohort (cross-sectional cohort) when they attended the Royal Free London NHS Foundation Trust (RFL) Department of Clinical Immunology for immunoglobulin infusions or clinic appointments; these patients provided a single sputum sample in a sterile container at each visit. The second cohort (prospective cohort) of RFL antibody-deficient patients were asked to provide samples every 2 weeks for 12 weeks and at the time they commenced additional antibiotics for presumed breakthrough infection. At each timepoint, they provided a sample in a sterile container for neutrophil elastase measurement and a sample in a microbiome preservation tube (Sputum DNA Collection, Preservation and Isolation Kit, Norgen Biotek, Canada). Clinical and demographic data were collected at each sample timepoint and symptoms were collected weekly for the prospective cohort.

All patients provided written, informed consent (NHS REC 04/Q0501/119).

### Sputum culture, organism identification and phenotypic antibiotic sensitivity

Sputum samples from the cross-sectional patient cohort were treated with dithiothreitol (Sputasol, Oxoid, UK) within 3–4 h of sample collection and 10 µL loopfuls were streaked on Columbia agar with chocolate horse blood agar (CHOC), cysteine lactose electrolyte-deficient (CLED) agar, Columbia colistin and nalidixic acid (can) agar, and Neomycin agar, (all culture media: Oxoid, UK) then incubated aerobically at 37°C with 5% carbon dioxide for 20–24 h. The remaining sputum samples were promptly stored at −70°C for molecular analyses.

All morphologically distinct colonies were purified and identified using MALDI-TOF MS (Bruker, Germany). Antimicrobial susceptibility of all isolates was tested by disc diffusion assays, following EUCAST guidelines.^11^ CLSI breakpoints were used when EUCAST lacked a corresponding value for the tested organism and antimicrobial agent.^12^ Bacterial isolates were stored in Microbank cryovials at −70°C for downstream analysis.

### DNA extraction

Frozen samples from the cross-sectional cohort or samples in microbiome preservation tubes from the prospective cohort were processed for DNA extraction. Five hundred microlitres of sample was centrifuged at 20 000 × **g** for 15 min, the supernatant was discarded and the pellet was heated at 95°C for 30 min, then mechanically disrupted by bead beating (FastPrep^®^ lysis matrix B, Fast-Prep^®^-24 Instrument; MP biomedicals, Germany) and enzymatically digested by proteinase K. The genomic DNA of 95 sputum isolates (8 *Haemophilus influenzae*, 6 *Pseudomonas aeruginosa*, 3 *Streptococcus pneumoniae* and 78 viridans-type *Streptococcus* species) was extracted using the DNeasy Blood and Tissue kit (QIAGEN, Germany) as per the manufacturer’s instructions. Metagenomic DNA was then extracted on the automated LIAISON^®^ Ixt extraction platform using the DiaSorin^®^ Arrow DNA extraction kit (DiaSorin, Ireland) as per the manufacturer’s protocol and eluted into 100 µL. The DNA was quantified using the Qubit dsDNA HS Assay kit.

### PCR for azithromycin resistance genes

Extracted DNA was amplified in a multiplex PCR for five representative genes conferring macrolide resistance [*erm*(A), *erm*(B), *erm*(C), *msr*(A) and *mef*(A)]. Primers and PCR conditions are provided in Table S1 (available as Supplementary data at *JAC-AMR* Online).

### Elastase measurement

Sputum samples were centrifuged at 18 000 × g for 1 h at 4°C and supernatants stored at −80°C. Neutrophil elastase was measured by the Protease Tag immunoassay (ProAxsis, UK) following the manufacturer’s instructions.

### Classification of antibiotic course appropriateness

Two authors (D.M.L. and T.M.) independently assessed the appropriateness of antibiotics taken by patients in the prospective cohort, with disagreements resolved by discussion to reach consensus. Antibiotic courses were deemed ‘appropriate’ (change in microbiome and rise in elastase), ‘late’ (appropriate but taken at least 2 weeks after change in microbiome/elastase) or ‘inappropriate’ (stable microbiome and no change in elastase). If no antibiotics were taken for episodes of elastase rise and microbiome change, this was termed ‘missed’.

### Viral PCR

Virus-specific PCRs were performed on the TaqMan 7500 (Applied Biosystems) using a one-step reverse transcription PCR kit; Power SYBR Green RNA to Ct 1-step Master Mix (Thermo Fisher Scientific), according to the manufacturer’s instructions. Each reaction was performed in a total volume of 20 µL and included previously validated primers specific for the viral target (see Table S2). Each assay included a negative water control, a ‘no template’ control and a positive control for the specific viral target. Amplification of a human target gene was also included to confirm absence of PCR inhibition. Cycle threshold (C_t_) values were compared with expected positive control values before PCR results were considered valid.

### 16S rRNA sequencing, WGS of bacterial isolates and metagenomic analysis

For 16S rRNA profiling, a sequence library was created by amplification of V3–V4 regions of the 16S rRNA gene through conventional PCR using 341 forward primers and 805 reverse primers fused with Nextera XT index and MiSeq adapter sequences (Sigma–Aldrich). Further PCR details are provided in the Supplementary Methods.

Sequencing was performed on the Illumina MiSeq Platform (Illumina, Inc., San Diego, USA) using MiSeq^®^ Reagent Kit v2 (500 cycles), which produces paired-end reads 2 × 250 bp, as per standard Illumina protocol. Bioinformatic analysis of the 16S rRNA data was done with the QIIME pipeline v.2.^13^ Taxonomic classification of the amplicon sequence variants was completed using the HOMD extended database. Taxonomy bar plots were generated using the 20 most abundant genera across all samples. α-Diversity indices were calculated on a rarefied feature table to a depth of 10 900 reads.

The WGS library of 95 bacterial isolates was prepared using the NEB Next Ultra II DNA library Prep kit (New England Biolabs, UK). Sequencing was performed on the Illumina NextSeq 500 platform (Illumina, Inc, San Diego, USA) using the Mid output Kit v2, which produces paired-end reads 151 bp as per standard Illumina protocol. Sequence data are deposited in the European Nucleotide Archive (ENA), accession number PRJEB65458.

As some isolates, especially the streptococci, were a mixture of closely related species, a metagenomics bioinformatic analysis approach was adopted as per the EBI-MGnify resource.^14^ Further details are provided in the Supplementary Methods.

Statistical analysis was performed using IBM SPSS Statistics, v.25.0.^15^ Two-tailed significance was set at *P* < 0.05.

## Results

### Patient characteristics

In the cross-sectional cohort, we collected 70 samples from 29 patients (range 1 to 6, median 2 samples per patient). The most common diagnosis was CVID (*n* = 18) and most had known bronchiectasis. Nineteen of 29 patients were on long-term antibiotic prophylaxis, of whom 11 were receiving macrolides [azithromycin (9/11) or clarithromycin (2/11)]. The prospective cohort comprised 14 patients. Again, CVID was the most common diagnosis; 12 of 14 had known bronchiectasis and 8 of 14 were on antibiotic prophylaxis. Previous positive bacterial cultures were relatively common in this group. Patient characteristics are summarized in Table 1.

**Table 1.** Patient characteristics

Characteristic		Cross-sectional cohort	Prospective cohort
Age (years)	Median	62	58
	Range	23–87	24–72
Sex	Male	14	8
	Female	15	6
Diagnosis	Common variable immunodeficiency	18	9
	X-linked agammaglobulinaemia	1	3
	Specific antibody deficiency	2	1
	Good’s syndrome	1	0
	Secondary antibody deficiency^a^	7	1
Antibiotic prophylaxis	Macrolide	11	6 ^c^
	Other antibiotic^b^	9	3 ^c^
	Nil	9	6
Frequency of breakthrough antibiotic usage	At least once a month	0	0
	Less than once a month but at least 6 times per year	1	0
	About 4 or 5 times per year	4	5
	About 2 or 3 times per year	10	7
	About once a year	3	2
	Less than once a year	4	0
	Unknown	7	0
Known bronchiectasis	Yes	21	12
	No	8	2
Positive microbiological results in respiratory samples in previous 12 months	*Rhinovirus*	2	0
	*H. influenzae*	2	5
	*S. pneumoniae*	0	1
	*Moraxella catarrhalis*	0	1
	*Staphylococcus aureus*	0	1
	*P. aeruginosa*	0	1

^a^Follicular lymphoma (2), myeloma (1), monoclonal gammopathy of uncertain significance (1), rheumatoid arthritis (1), granulomatosis with polyangiitis (1), sarcoidosis with lung transplant (1).

^b^Cross-sectional cohort: amoxicillin (1), penicillin V (1), ciprofloxacin (1), co-trimoxazole (2), trimethoprim (1), cefalexin (1), doxycycline (1). Prospective cohort: co-trimoxazole (1), ciprofloxacin (1), doxycycline (1)

^c^The patient receiving doxycycline prophylaxis also took daily azithromycin and is counted in both categories.

### Phenotypic antibiotic resistance is common in patients with antibody deficiency

**Figure 1.**
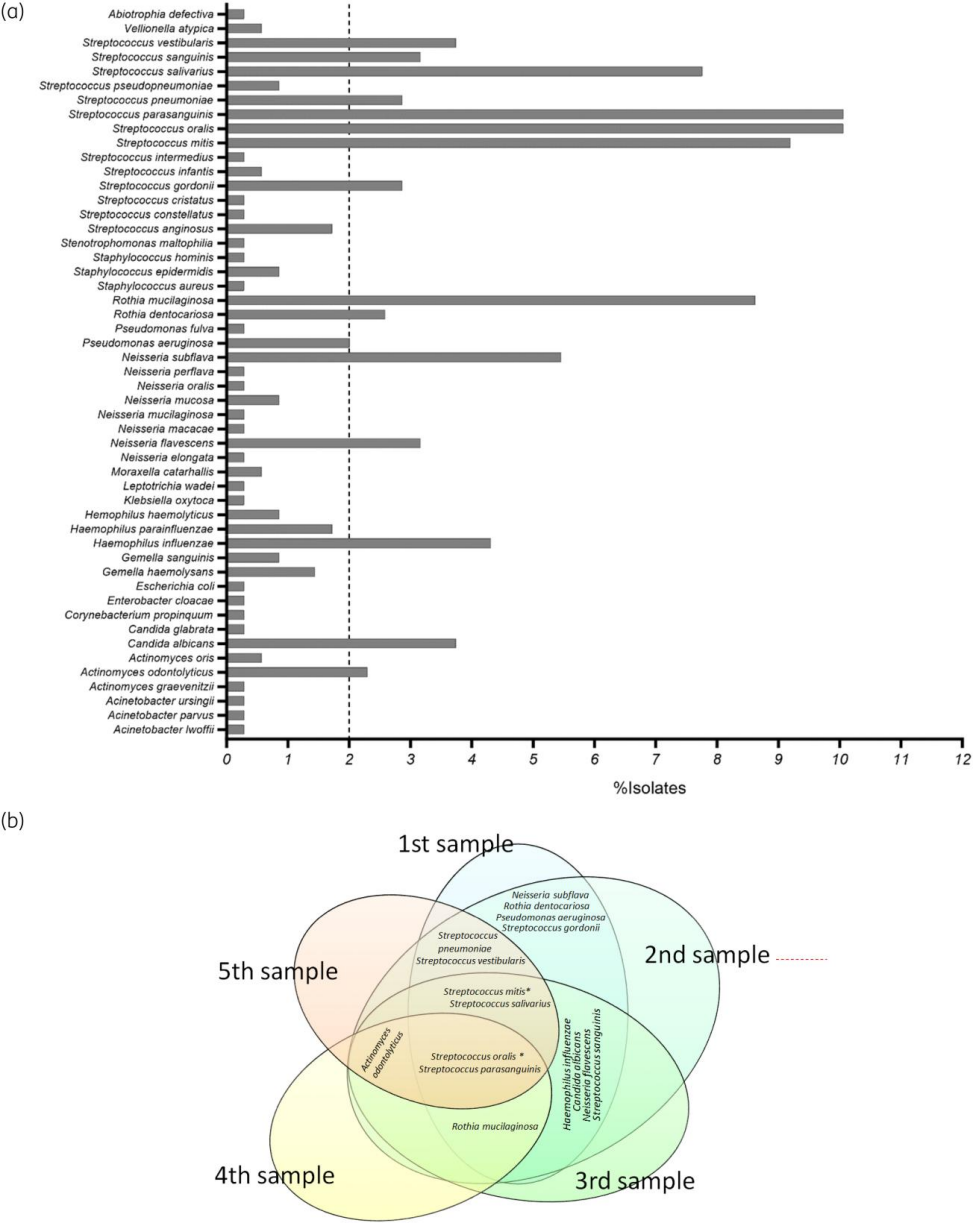
(a) Seventy sputum samples were collected from patients with antibody deficiency and cultured on agar plates. Visible colonies were identified to species level via MALDI-TOF and are presented here (*n* = 343). Species present in at least 2% of samples (dotted line) were further analysed for phenotypic antibiotic resistance. (b) Venn diagram indicating organisms shared between samples in those patients who provided more than one sample.

All visible colonies (*n* = 343) on agar plates from culture of 70 sputum samples from the cross-sectional cohort were speciated using MALDI-TOF; organisms and frequencies are presented in Figure 1(a). In patients who provided more than one sample, the microbial community was largely stable, especially for commensal streptococci (Figure 1b).

**Figure 2.**
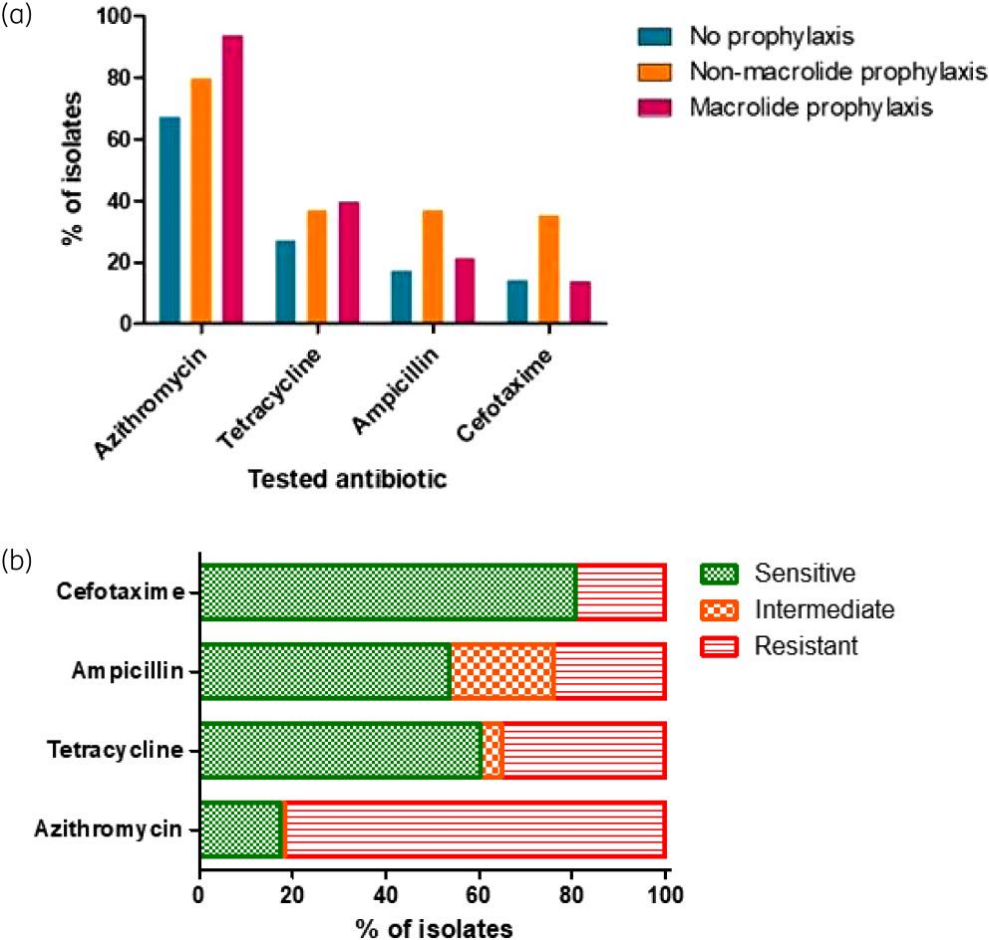
(a) Percentage of 255 isolates (*Streptococcus* spp. or *H. influenzae*) with phenotypic resistance to various antibiotics as assessed by disc diffusion, arranged according to the patients’ antibiotic prophylaxis. (b) Overall susceptibility to the four antibiotics across all isolates.

We proceeded to assess phenotypic antibiotic sensitivity in any species that constituted at least 2% of isolates. Given intrinsic resistance among some organisms, we present summary data for 255 isolates of *Streptococcus* spp. (including *S. pneumoniae*) and *H. influenzae* (Figure 2). Azithromycin resistance was particularly prevalent, even among those patients not receiving macrolide prophylaxis. Overall, 81.7% of isolates were resistant to azithromycin, 35.0% resistant to tetracyclines, 23.9% resistant to ampicillin and 19.3% resistant to cefotaxime.

### Macrolide resistance genes are commonly present in sputum samples, even in patients not receiving macrolide prophylaxis

**Figure 3.**
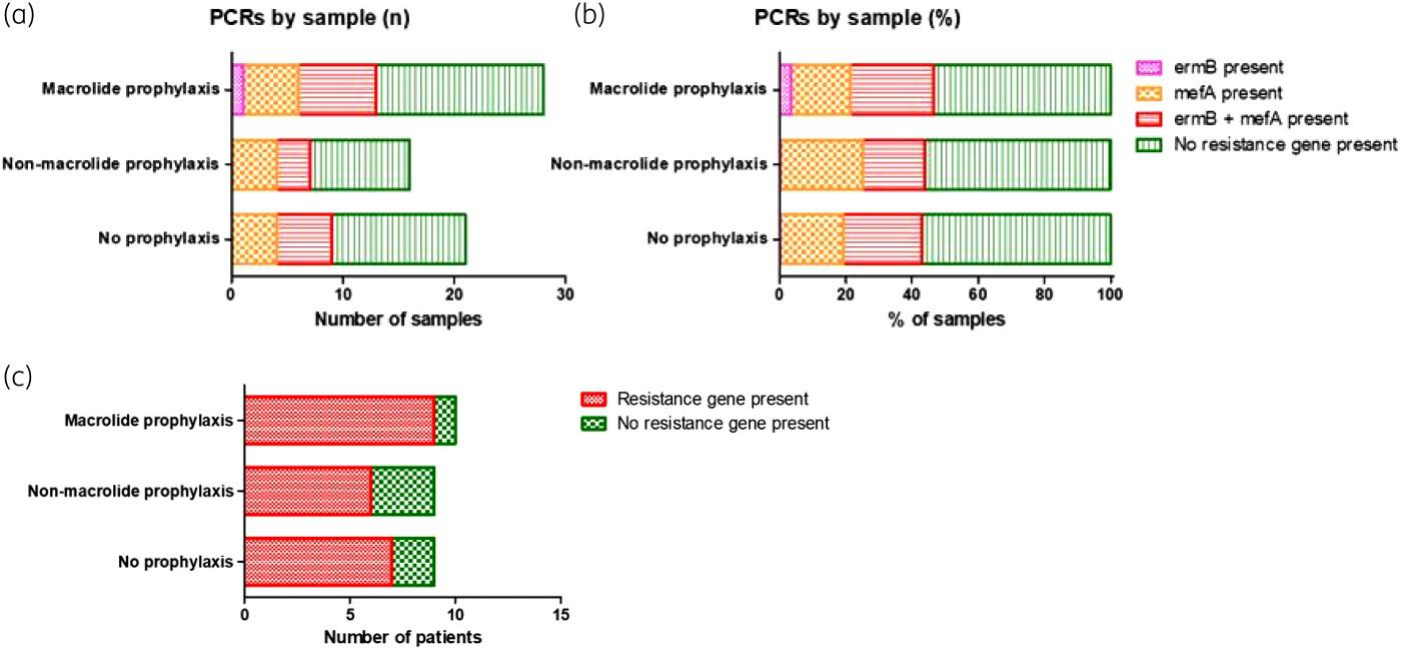
(a and b) Presence of individual macrolide resistance genes [*erm*(B) and *mef*(A)] in sputum samples, presented according to patients’ antibiotic prophylaxis, as absolute number (a) or percentage (b) of samples. (c) Patient-level data indicating presence or absence of any macrolide resistance gene in any sample, presented according to patients’ antibiotic prophylaxis.

In view of the high rate of phenotypic azithromycin resistance, we analysed 65 sputum samples (from 28 patients) for the presence of macrolide resistance genes by PCR, finding *erm*(B) and/or *mef*(A) present in over 40% of samples, regardless of the type of prophylaxis (Figure 3a and b). As some patients provided more than one sample, we also analysed per patient and observed that only 6/28 patients (1/10 on azithromycin prophylaxis, 3/9 on other prophylaxis and 2/9 on no prophylaxis) had no samples positive for these resistance genes (Figure 3c). PCRs for the other tested resistance genes (Table S1) were negative.

### Bacterial sequencing demonstrates extensive molecular evidence of antibiotic resistance and possible transmission between patients

**Figure 4.**
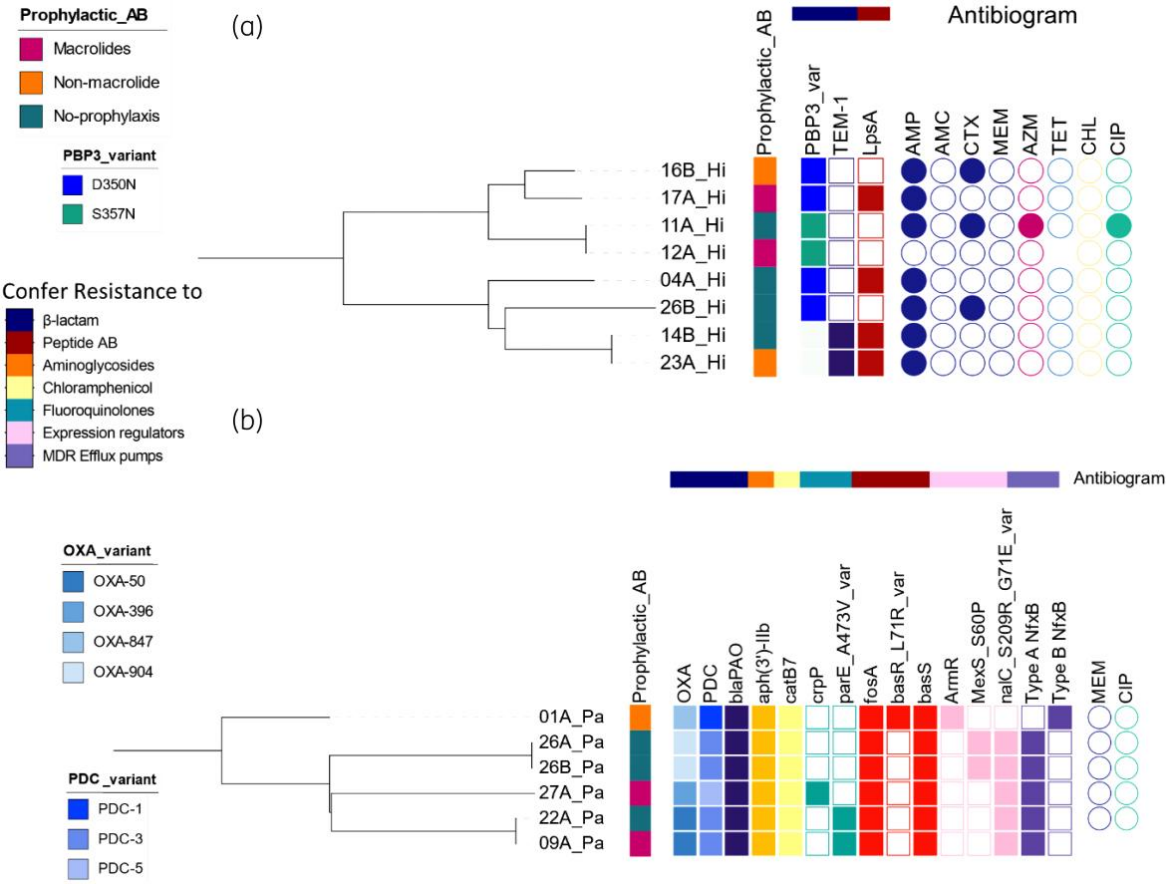
The clustering, resistome data and antibiogram of the sequenced Gram-negative isolates. (a) *H. influenzae* isolates (*n* = 8). (b) *P. aeruginosa* isolates (*n* = 6). Each row represents one isolate and is labelled by: the patient study number, a letter indicating the sample (A for the first sample submitted by that patient, B for the second) and an abbreviation for the bacterial species (Hi, *H. influenzae*; Pa, *P. aeruginosa*). Antibiotic prophylaxis therapy status of the corresponding participants is represented by the first column of coloured squares. The detected AMR genes are represented by solid shaded squares; the colours of the heat map correspond to the detected variant of the genes (for PBP3, OXA and PDC) or the class of antibiotics they confer resistance to (also represented by the colour band above heat map). Phenotypic resistance or intermediate susceptibility from disc diffusion tests are represented by solid shaded circles in the antibiogram: AMP, ampicillin; CTX, cefotaxime; MEM, meropenem; AZM, azithromycin; TET, tetracycline; CHL, chloramphenicol, CIP, ciprofloxacin.

From cluster analysis (Figure 4), two pairs of *H. influenzae* isolates, each from different patients, were phylogenetically close and clustered into two clades on the tree (Figure 4a). Similarly, two pairs of *P. aeruginosa* isolates were phylogenetically close; the first pair were serial isolates from the same patient over a 4 week period, but the second pair were from different patients (Figure 4b).

Figure 4(a) further details the resistome of the *H. influenzae* isolates (*n* = 8). The functions and mechanisms of the AMR genes are detailed in Table S3. TEM-1 was detected in two isolates. Two different variants of PBP3 were detected in the other six isolates: D350N in four and S357N in two. *lpsA* was detected in four of the isolates. No plasmids or mobile genetic elements were detected in any *H. influenzae* isolate. All ISs detected were imperfect hits (sequence identity <90% and/or alignment coverage <95%).

In all *P. aeruginosa* isolates (*n* = 6), as shown in Figure 4(b), the following antibiotic resistance determinants were detected: the β-lactamase-encoding genes *bla*_PAO_, *bla*_OXA_ and *bla*_PDC_ (the exact variants of the latter two as per the CARD database^16^ are shown by a colour strip in Figure 4). In addition, *aph(3′)-IIb*, *fosA* and *catB7* were detected in all tested *P. aeruginosa* isolates.

Determinants of resistance to cationic antimicrobial peptides (e.g. colistin and polymyxin), *arnA* and *cprRS*, were detected in all *P. aeruginosa* isolates. The regulatory system *basRS* (*pmrAB)* was also identified; *basS* was detected in all isolates while the *basR* with L71R mutation was detected in only one isolate.

The following multidrug efflux pump systems and their regulators were detected in all *P. aeruginosa* isolates apart from the bold-faced moieties, which were not detected in occasional isolates as specified: *mexA**B**-oprM* (*mexB* was not detected in two samples) and its regulators *cpxRA*, *acrD*, *nalD* and *mexR*; *mexCD-oprJ*, *mexE**F**-oprN* (*mexF* not in two samples) and its regulators *rsmA*, *mexS* and *mexT*; *mexGHI-opmD* and its regulator *soxR*; *mexLJK-opmH*; *mexMN-oprM*; *mexPQ-opmE*; *mexVW-oprM*; *mexXY-oprM* and its two-component sensor *parRS*; *muxABC-**opmB*** (*opmB* not in one sample); *acrAB-tolC* and its regulator *yajC*; *pmpM*; *emrE* and *triABC-opmH*.

The following AMR genes were detected in some *P. aeruginosa* isolates (as shown by the heat map in in Figure 4b): *crpP*, *parE* with the point mutation A473V, *armR*, *mexS* with point mutation S60P,^17^  *nalC* with S209R and G71E mutations.

In all *P. aeruginosa* isolates except one, *fosA* was linked to ISs such as ISPa1 and ISPa2. Other insertion sequences like ISPa6, ISPa22, ISPa32, ISPa37, ISPre2, ISPsy29 and ISPa45 were also detected but not linked with AMR genes. A unit transposon Tn*5563* was detected in one isolate. No plasmids were identified in the tested *P. aeruginosa*.

**Figure 5.**
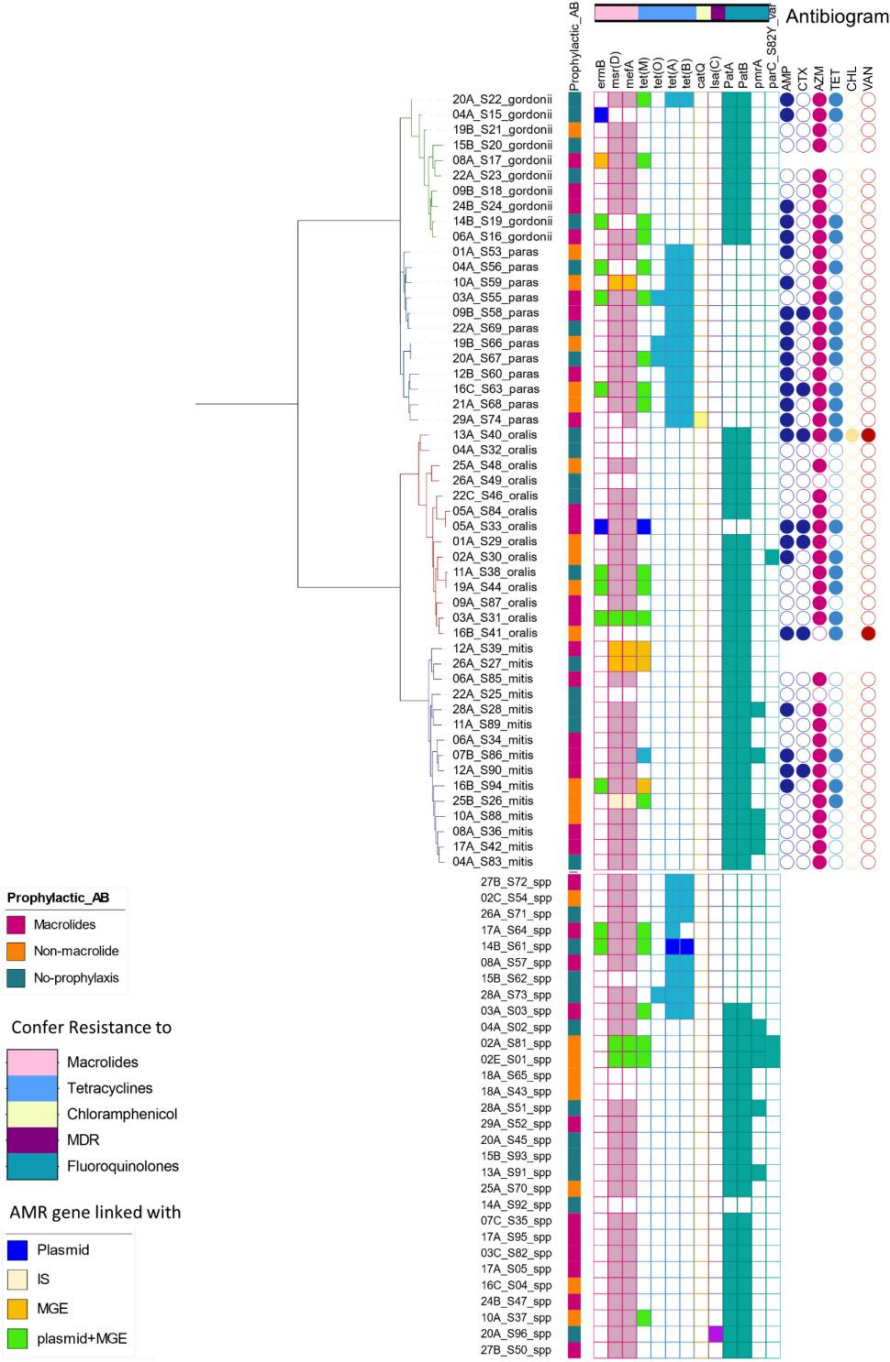
The clustering, resistome data and antibiogram of the sequenced streptococci isolates. (a) The first clade (green branches), *S. gordonii* isolates. (b) Second clade isolates (blue branched), *S. parasanguinis*. (c) Third clade (red branches), *S. oralis*. (d) Fourth clade (purple branches), *S. mitis*. (e) A mixture of closely related *Streptococcus* species. Each row represents one isolate and is labelled by: the patient study number, a letter indicating the sample (A for the first sample submitted by that patient, B for the second etc.), an isolate number in the form Sn and the streptococcal species (where paras = *parasanguinis*). Antibiotic prophylaxis therapy status of the corresponding participants is represented by the first column of coloured squares. The detected AMR genes are represented by solid shaded squares, the colours of the heat map correspond to how the AMR genes are linked to: plasmids, ISs, or mobile genetic elements (MGEs) (unit transposon, composite transposon or ICE), or the class of antibiotics they confer resistance to (also represented by the colour band above the heat map). Phenotypic resistance or intermediate susceptibility from disc diffusion tests are represented by solid shaded circles in the antibiogram: AMP, ampicillin; CTX, cefotaxime; AZM, azithromycin; TET, tetracycline; CHL, chloramphenicol; VAN, vancomycin, _var: variant.

Fifty-one Gram-positive isolates (Figure 5) were classified according to the core genome k-mers as: 10 *Streptococcus gordonii*, 12 *Streptococcus parasanguinis*, 14 *Streptococcus oralis* and 15 *Streptococcus mitis*. However, 30 isolates were mixed cultures of closely related *Streptococcus* species. These are grouped separately at the end of Figure 5: note that this included three *S. pneumoniae* isolates, which were mixed with *Streptococcus pseudopneumoniae.* Resistome analysis of the tested streptococci isolates (Figure 5) revealed that *erm*(B), *msr*(D) and *mef*(A) were the most common determinants of macrolide resistance. *erm*(B) was detected in 16% of isolates (*n* = 13), 92% (*n* = 12) of them linked with plasmid repUS43; in addition, 85% (*n* = 11) were also linked with integrative conjugative elements (ICEs) such as Tn*6009*, Tn*916* and Tn*917*. Also, in 85% (*n* = 11), *erm*(B) existed in conjugation with *tet*(M) genes on repUS43 plasmids. Both *msr*(D) (*n* = 68; 84%) and *mef*(A) (*n* = 69; 85%) genes were identified in the isolates, and in most cases (98.6%) both genes were detected in close proximity. Both *lsaC* and *cat(Q)* were detected once in two different resistomes.

The *tet* genes *tet*(M), *tet*(O) and *tet*(A/B) were the most common determinants of tetracycline resistance, detected in 24 (30%), 4 (5%) and 21 (26%) of the streptococcal isolates, respectively. In 96% of the isolates in which *tet*(M) was detected, it was linked with plasmids (repUS43) and/or ICE [Tn*6009* or ICESpn11928 (family Tn*916*)].

The CARD database^16^ identified fluoroquinolone resistance determinants. *patA/B* was found in 72% (*n* = 58) of the tested streptococci but only half were perfect hits (identity > 95%, coverage 100%). Of these, 17% (*n* = 10) had *pmrA* in addition.

**Figure 6.**
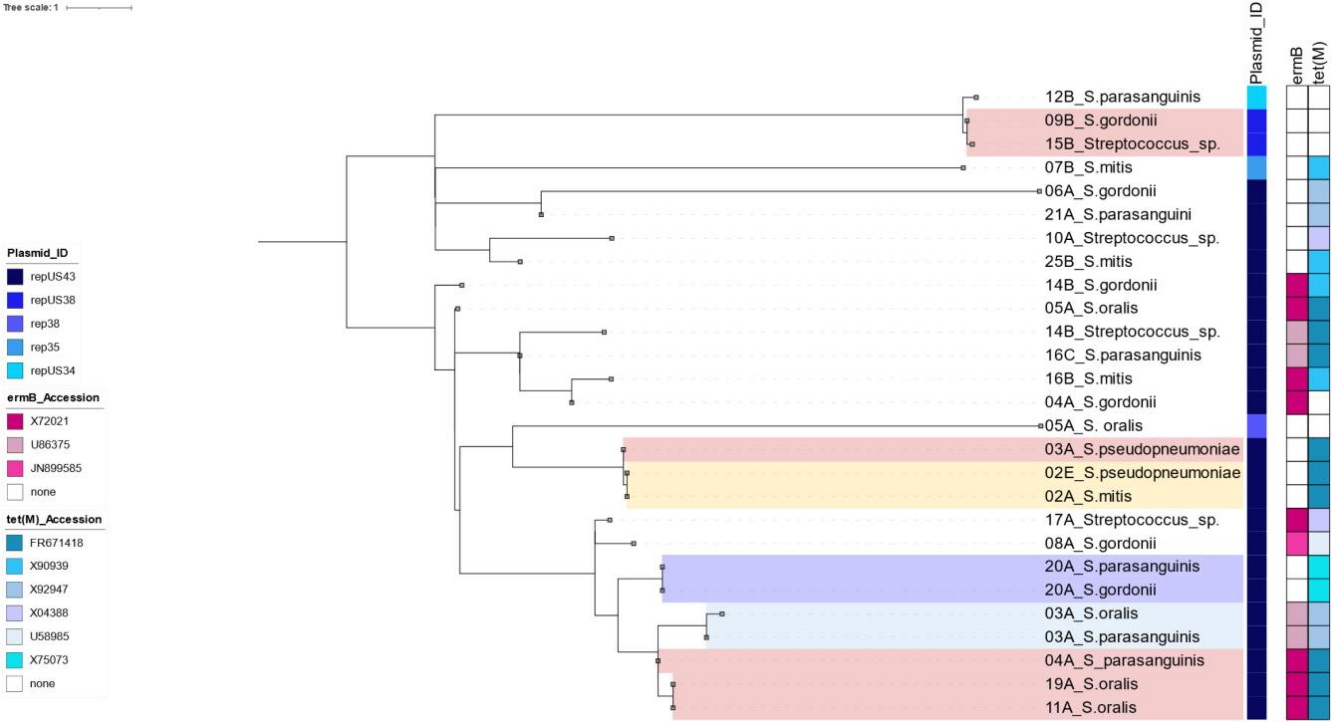
Clustering analysis of plasmid-derived sequences and their linkage with AMR genes that confer resistance to macrolides and tetracyclines. The clades highlighted pink are isolates from different patients, the yellow highlighted clade is pair of different streptococci isolates from serial sputum samples of the same patient, the blue highlighted clades are pairs of different streptococci isolates from the same sputum samples.

Phylogenetic analysis on extracted plasmid sequences demonstrated that conjugative plasmids such as repUS43 and repUS38, which may carry *erm*(B) and/or *tet*(M) genes, clustered in clades including different streptococcal isolates from the same sputum sample or serial sputum samples from the same patient and even between different patients (Figure 6), which may suggest cross-transmission.

### Prospectively collected samples demonstrate a relationship between changes in sputum elastase and bacterial α-diversity

Fourteen patients provided serial sputum samples for measurement of elastase and 16S rRNA microbiome. There was a significant negative relationship between neutrophil elastase concentration and markers of α-diversity: Shannon entropy α-diversity index (Spearman’s ρ = −0.306, *P* = 0.005) and evenness indices [Simpson-e (ρ = −0.510, *P* < 0.001) and Pielou evenness index (ρ = −0.651, *P* < 0.001)], but a positive relationship with indicators of microbial dominance [e.g. Berger–Parker dominance index (ρ = 0.502, *P* < 0.001)] (Table 2). We also calculated the change in elastase from one timepoint to the next in each patient and correlated this with the corresponding change in α-diversity metrics; again, we observed significant negative correlations with markers of richness and evenness but a positive relationship with dominance, implying that an increase in elastase from one sample to the next predicted a reduction in α-diversity (Table 2).

**Table 2.** Correlations between elastase concentration (or change in elastase concentration between consecutive samples) with corresponding α-diversity metrics

Metric	Elastase concentration versus α-diversity metric		Change in elastase concentration versus change in α-diversity metric between consecutive samples	
	Spearman rho	*P* value	Spearman rho	*P* value
Shannon entropy	−0.306	0.005	−0.273	0.034
Simpson e	−0.651	<0.001	−0.287	0.017
Pielou evenness index	−0.510	<0.001	−0.242	0.046
Berger–Parker dominance index	0.502	<0.001	0.302	0.012

We also looked for correlations between abundance of the most common taxa and elastase. We observed a significant positive relationship between *H. influenzae* abundance and elastase concentration (ρ = 0.244, *P* = 0.027) and a negative relationship between *Streptococcus* spp. and elastase (ρ = −0.469, *P* < 0.001). This implies that elastase concentration is increased in the presence of pathogens and relatively lower when there is a greater abundance of commensal streptococci.

Nine patients took 11 courses of antibiotics during the study period. On the basis of their elastase and microbiome analysis, we classified 5 of these courses as appropriate (see, for example, Figure S1a), 4 courses as inappropriate due to no change in elastase or microbiome (Figure S1b) and 2 courses as appropriate but taken late (Figure S1c). We also identified four potential missed opportunities for antibiotics (Figure S1d).

### Neutrophil elastase concentrations are low in samples testing positive for pathogenic viruses

Viral PCRs were positive in six samples. Rhinovirus was detected in five samples from one patient; of note, they took antibiotics despite low elastase concentration and no change in microbiome (Figure S1b). One sample from another patient was positive for human metapneumovirus; again, neutrophil elastase concentration was low at this timepoint.

## Discussion

Patients with antibody deficiency commonly take prophylactic antibiotics;^9^ for secondary antibody deficiency, UK commissioning guidelines require this therapy for 6 months prior to consideration of immunoglobulin replacement therapy.^18^ These patients also frequently receive antibiotic treatment courses for breakthrough infection.^5^ Although we have previously demonstrated chronic inflammation and dysbiosis in the airways of similar patients,^8^ the cumulative impact of these antibiotic interventions on antimicrobial resistance has not been well studied.

Here, we have demonstrated that respiratory tract bacteria from patients with antibody deficiency exhibit extremely high rates of antibiotic resistance. This was evident phenotypically using standardized disc diffusion tests as well as via molecular techniques (both targeted PCR and genomics). Multidrug efflux pumps are frequently detected in pathogens such as *P. aeruginosa*. These can contribute to the observed MDR phenotypes and the persistence of these bacteria in the airways of this patient population. However, for other bacteria, much of the observed resistance is likely to be driven by therapeutic use of antibiotics. Resistance to macrolides was particularly prevalent, consistent with this class being most commonly used as prophylaxis, and interestingly was seen at high levels even in patients not currently receiving azithromycin or clarithromycin. This may reflect historical usage of these antibiotics or transfer of resistance genes between individuals. Resistance was also common to other antibiotic classes, especially tetracyclines and, to a lesser extent, β-lactams.

The majority (22/28) of patients had a positive PCR for the macrolide resistance genes *erm*(B) or *mef*(A*)* in at least one sample. Sequencing of individual bacterial isolates localized these, as well as genes conferring resistance to tetracyclines, fluoroquinolones and other antibiotics, predominantly to commensal streptococcal species. These species have been demonstrated to harbour these genes previously^19^ and it has been proposed that they can act as a reservoir for horizontal transmission to pathogenic species.^20^

In addition to horizontal transmission between bacterial species, organisms may be transferred between hosts, especially as these patients often share the same clinical space for immunoglobulin infusions.^10^ Sequencing identified several pairs of almost identical pathogens (*H. influenzae* and *P. aeruginosa*) in samples from different individuals. We cannot definitively prove cross-transmission or, if transmission had occurred, that this was within the hospital. However, this is a likely explanation and requires further investigation. If transmission of commensal bacteria is also occurring, horizontal transfer of resistance genes might occur to another patient; this may help to explain high rates of antibiotic resistance even in people not taking prophylaxis.

The high prevalence of antibiotic resistance demands strategies to minimize antibiotic usage. Our previous research has suggested that ‘respiratory exacerbations’ in patients with antibody deficiency are more often positive for pathogenic viruses than bacteria.^5^ Patients self-treated for presumed bacterial infection even with predominantly upper respiratory tract symptoms, but response to antibiotics was predicted by sputum purulence.^5^ We therefore investigated the relationship between neutrophil elastase and bacterial populations in sputum as a rapid tool to inform antibiotic use.

There were strong negative relationships between sputum elastase concentration and markers of α-diversity or evenness but positive relationships with markers of dominance. This is similar to previous findings in a cross-sectional study.^8^ Importantly, there were similar relationships between the change in elastase from one sample to the next and the change in microbiome metrics. Thus, even within individual patients, a rise in neutrophil elastase concentration is associated with a reduction in α-diversity with an increase in bacterial dominance, consistent with infection or bacterial exacerbation. There was also a positive relationship between neutrophil elastase concentration and abundance of the pathogen *H. influenzae* but a negative relationship with the abundance of streptococci (most of which are commensals).^20^ Conversely, samples that tested positive for pathogenic viruses exhibited low elastase concentrations.

Collectively, these results suggest that sputum neutrophil elastase concentration is a useful indicator of dysbiosis and potentially of acute bacterial infection. Prospective data on elastase and 16S rRNA microbiome indicated that some patients are probably using antibiotics inappropriately while others are taking antibiotics late or not at all, even when they are likely to be indicated. Given the role of neutrophil elastase in driving lung damage such as bronchiectasis,^7,21^ prompt treatment should be directed at bacterial exacerbations associated with a rise in elastase. Prospective monitoring of neutrophil elastase concentration may be a useful guide for antibiotic treatment decisions. Ultimately, we envisage a point-of-care test for sputum neutrophil elastase concentration to help patients and clinicians with antibiotic choices.

Our study has limitations. The cohorts were relatively small, although this largely reflects the rarity of the underlying conditions, especially as patients were also required to spontaneously produce sputum. This was a single-centre study, which may limit applicability of results to all settings, although practice with respect to antibiotics is generally similar elsewhere.^9^ Some of the species identified among the commensal streptococci by MALDI-TOF did not perfectly align with the results from sequencing, and occasionally sequencing identified a mixed population, but all were within the viridans group. Targeted PCR was only performed for two resistance genes but metagenomic data complemented this with numerous other targets. WGS of the isolates was performed using Illumina (a short-read sequencing technology, which produce 150 bp reads); although Illumina provides high-accuracy sequencing, the short reads have limited the construction of plasmids and mobile genetic elements.

In summary, we have demonstrated high levels of antibiotic resistance in the respiratory tract microbiota of patients with antibody deficiency. This is especially prevalent for macrolides, which are commonly used as long-term prophylaxis. We recommend reassessment of the long-term clinical utility of antibiotic prophylaxis, especially for patients already receiving immunoglobulin replacement, and investigation of alternative prophylactic agents. Metagenomic analysis confirms a complex resistome with commensal streptococci possibly acting as reservoirs for resistance genes, which can be transferred to other bacteria within the microbiome or disseminated into the environment and other hosts. Patients appear to transmit organisms between each other, and horizontal transfer of resistance genes may occur between species and hosts. This has implications for the management of patients, suggesting home administration of immunoglobulin replacement therapy where possible, minimization of close contact between patients in healthcare facilities and strict infection control practices. It is also feasible that immunocompromised, antibiotic-experienced individuals act as a source of resistant organisms in the wider community, although we did not test this hypothesis. Finally, neutrophil elastase concentration in sputum might be a useful biomarker to guide antibiotic usage, perhaps via a point-of-care test, ensuring that these precious medications are used appropriately.

## Supplementary Material

dlad135_Supplementary_Data

## Supplementary data

Figure S1 and Tables S1, S2 and S3 are available as Supplementary data at *JAC-AMR* Online.
