# Supplementary material for: Antibiotic resistance, bacterial transmission and improved prediction of bacterial infection in patients with antibody deficiency
Source: JAC Antimicrob Resist. 2023 Dec 14;5(6):dlad135. doi: 10.1093/jacamr/dlad135 (PMC10720947; doi:10.1093/jacamr/dlad135)
Supplement: dlad135_Supplementary_Data [file dlad135_supplementary_data.docx]

**Supplementary Material**

**Supplementary Methods**

**PCR for 16S rRNA sequencing**

PCR was carried out as follows: initial denaturation at 95˚C for 5 minutes, then 30 cycles of denaturation at 95˚C for 30 seconds, annealing at 55˚C for 40 seconds and extension at 72˚C for 60 seconds plus a final extension at 72˚C for 10 minutes. The PCR products (584 bp amplicons) were cleaned using Agencourt AMPure XP beads (Beckman Coulter, UK) and quantified using Qubit dsDNA HS Assay kit and the DNA fragments integrity was checked by TapeStation (Agilent, USA). The sequencing library was created by pooling the samples at equimolar ratio at final concentration of 5 nM.

**Metagenomics bioinformatic analysis approach for analysis of streptococci**

Briefly, the raw reads were trimmed off adaptors and low-quality terminal bases <20 phred score, then reads less than 50 bp were filtered out. De-novo assembly of the genomes was performed using SPAdes v.3.15.3^1^. The identity of the assembled contigs was confirmed using KmerFinder v.3.0.2^2^. Resistome was investigated by mapping the assembled contigs against the ResFinder v.4.1^3^ and CARD v.3.2.7-RGI v.6.0.2 database^4^. Putative plasmid sequences in streptococci isolates were determined using PlasmidFinder v.2.1^5^. Presence of mobile genetic elements and their linkage with antimicrobial resistance genes was investigated using MobileElementFinder v.1.0.3 and database v.1.0.2^6^. Clustering analysis among isolates of closely related species was performed using kSNP v. 4^7^. The trees were inferred using the parsimony method from the matrix of core genome SNPs, based on k-mer analysis of genome sequences of the tested isolates. Phylogenetic analysis was performed on filtered contigs containing plasmid-derived sequences in the tested streptococci isolates using kSNP4.

**References**

1. Nurk S, Meleshko D, Korobeynikov A, Pevzner PA. metaSPAdes: a new versatile metagenomic assembler. *Genome Res* 2017; **27**(5): 824-34.

2. Hasman H, Saputra D, Sicheritz-Ponten T, et al. Rapid whole-genome sequencing for detection and characterization of microorganisms directly from clinical samples. *J Clin Microbiol* 2014; **52**(1): 139-46.

3. Florensa AF, Kaas RS, Clausen P, Aytan-Aktug D, Aarestrup FM. ResFinder - an open online resource for identification of antimicrobial resistance genes in next-generation sequencing data and prediction of phenotypes from genotypes. *Microb Genom* 2022; **8**(1).

4. Alcock BP, Huynh W, Chalil R, et al. CARD 2023: expanded curation, support for machine learning, and resistome prediction at the Comprehensive Antibiotic Resistance Database. *Nucleic Acids Res* 2023; **51**(D1): D690-d9.

5. Carattoli A, Hasman H. PlasmidFinder and In Silico pMLST: Identification and Typing of Plasmid Replicons in Whole-Genome Sequencing (WGS). *Methods Mol Biol* 2020; **2075**: 285-94.

6. Johansson MHK, Bortolaia V, Tansirichaiya S, Aarestrup FM, Roberts AP, Petersen TN. Detection of mobile genetic elements associated with antibiotic resistance in Salmonella enterica using a newly developed web tool: MobileElementFinder. *J Antimicrob Chemother* 2021; **76**(1): 101-9.

7. Gardner SN, Slezak T, Hall BG. kSNP3.0: SNP detection and phylogenetic analysis of genomes without genome alignment or reference genome. *Bioinformatics* 2015; **31**(17): 2877-8.


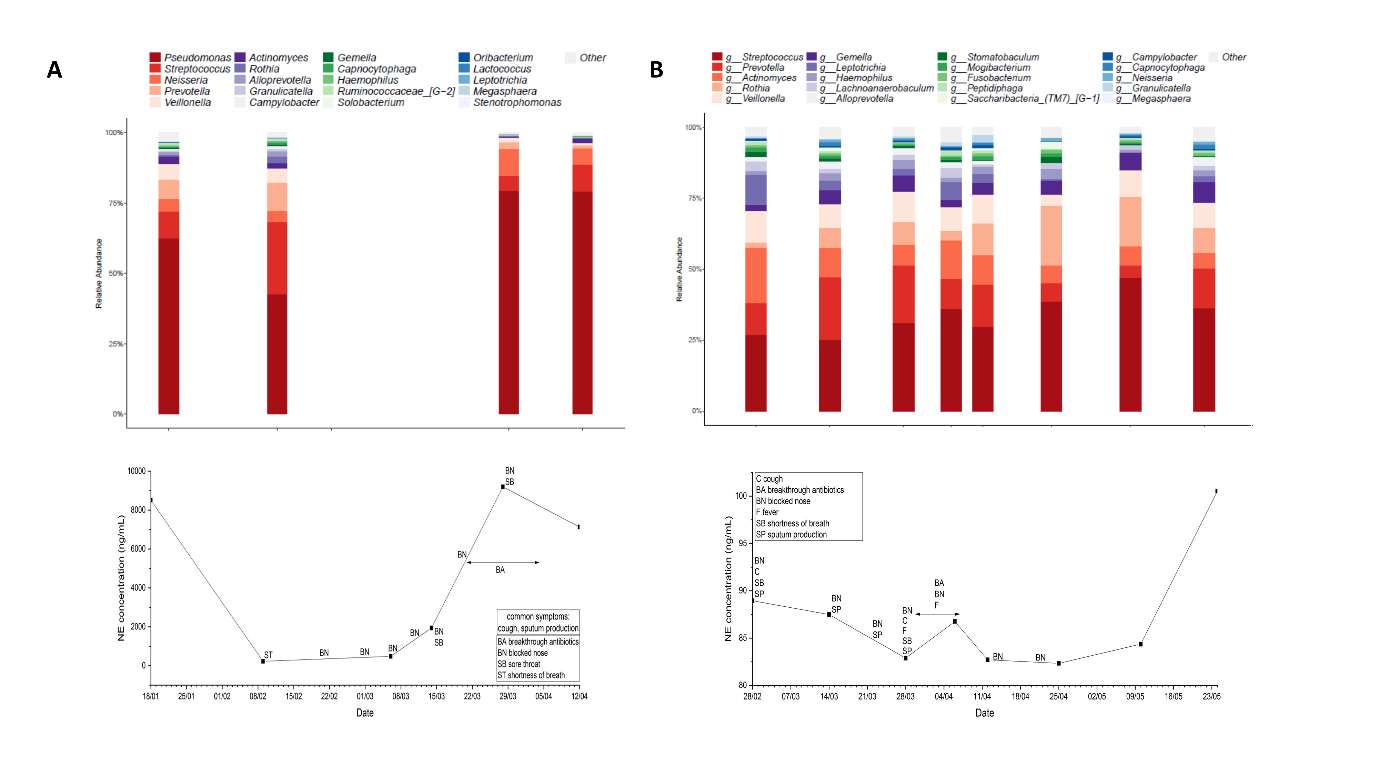


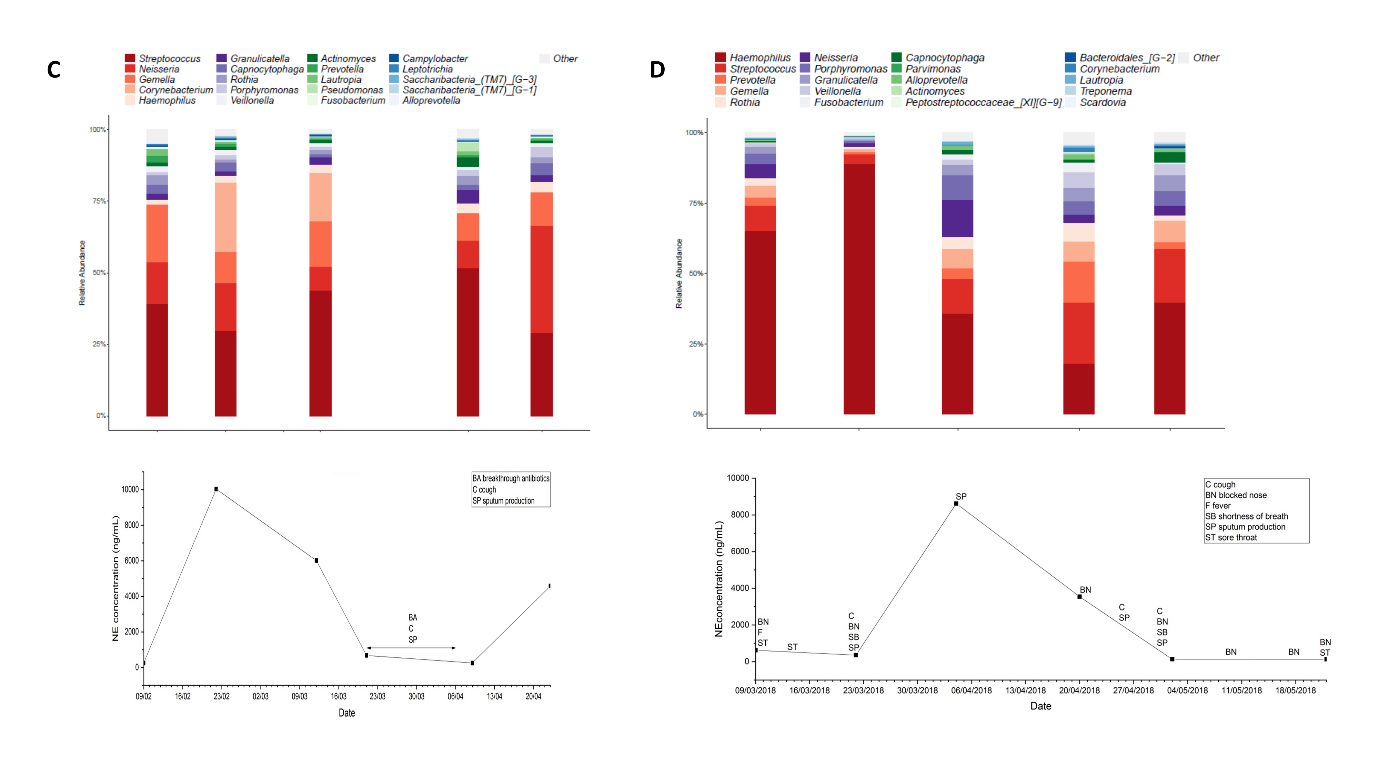


**Supplementary Figure 1.** Taxa bar charts and sputum neutrophils elastase (NE) concentrations over time. Elastase graphs are annotated with abbreviations indicating symptoms; BA = breakthrough antibiotics, course indicated by a horizontal line. **A.** Appropriate use of antibiotics corresponding with spike in elastase concentration and expansion of *Pseudomonas* abundance. **B.** Inappropriate use of antibiotics with no significant change in elastase concentration (note y-axis scale) or microbiome. Note that this patient’s samples tested positive for Rhinovirus in weeks 4, 5, 6, 10 and 12. **C.** Late intake of antibiotics following spike in elastase concentration and possible expansion of *Corynebacterium*. **D.** Potential missed opportunity for antibiotics with increase in *Haemophilus* abundance and subsequent spike in elastase concentration.

**Supplementary Table 1. Primers and PCR conditions for azithromycin resistance genes**

| ***Gene*** | **Genbank accession number** | **Sequence (5_-3_)** | **References** | **PCR conditions** |
| --- | --- | --- | --- | --- |
| *erm*(A) | X03216 | *CTT CGA TAG TTT ATT AAT ATT AGT*  *TCT AAA AAG CAT GTA AAA GAA* | Chung et al., 1999. | 94°C for 30 s, 48°C for 1 min, 72°C for 2 min |
| *erm*(B) | X52632 | *GAA AAA GTA CTC AAC CAA ATA*  *AGT AAT GGT ACT TAA ATT GTT TAC* | Chung et al., 1999. | 94°C for 30 s, 50°C for 30 s, 72°C for 2 min, 35 cycles |
| *erm*(C) | M19652 | *GCT AAT ATT GTT TAA ATC GTC AAT*  *TCA AAA CAT AAT ATA GAT AAA* | Chung et al., 1999. | 94°C for 30 s, 43°C for 1 min, 72°C for 2 min |
| *msr*(A) | X52085 | *GGC ACA ATA AGA GTG TTT AAA GG*  *AAG TTA TAT CAT GAA TAG ATT GTC CTG TT* | Lina et al., 1999. | 1 min at 94°C, 1 min at  50°C, 90 s at 72°C, 25 cycles |
| *mef*(A) | U70055 | *GGA CCT GCC ATT GGT GTG*  *ACC GAT TCT ATC AGC AAA G* | Luna et al., 2000. | 94°C for 1 min, 37°C for 1 min, 72°C for 2 min, 35 cycles |

**Supplementary Table 2. Primer and probe sequences for each of the viral targets detected by the Real‐Time Respiratory RT‐PCR assay**

| **Target** | **Primer name** | **Sequence (5’ to 3’)** | **Final concentration (nM)** | **Reference** |
| --- | --- | --- | --- | --- |
| Influenza A (matrix) | Forward Reverse | CAAGACCAATCCTGTCACCTCTG TGCATTTTGGACAAAGCGTCTAC | 900 900 | Bibby et al 2011 |
| Influenza B (haemaglutinin) | Forward Reverse | AAATACGGTGGATTAAATAAAAGCAA CCAGCAATAGCTCCGAAGAAA | 300 300 | Van Elden et al 2001 |
| Parainfluenza 1 (nucleoprotein) | Forward Reverse | ATTCAGACAGGATGGAACCGTYAA GATACTAAGCTTTGTTGTGACCTCAT | 900 900 | Modified from Bibby et al 2011 |
| Parainfluenza 2 (nucleoprotein) | Forward Reverse | AGAGATAACAGGGTTTGAGAATAATTCAT CAAATGGAGTTTGGTGATTAAGGGTA | 900 900 | Bibby et al 2011 |
| Parainfluenza 3 (nucleoprotein) | Forward Forward 2 Reverse | CGATTAGAGGCTTTCAGACAAGATG CGATTGGAAGCTTTCAGACAAGAYG | 900 900 | Modified from Bibby et al 2011 |
| Parainfluenza 4 (Nuceloprotein) | Forward Reverse | CAAAYGATCCACAGCAAAGATCC ATGYGGCCTGTAAGGAAAGCA | 900 900 | Van der Pol 2007 |
| Respiratory Syncytial Virus (Matrix) | Forward Reverse 1 Reverse 2 | GGAAACATACGTGAACAAGCTTCA CATCGTCTTTTCTAAGACATTGTATTGA TCATCATCTTTTCTAGAACATTGTACTGA | 900 900 900 | Kuypers et al 2004 |
| Adenovirus (Hexon) | Forward Reverse | GCCCCAGTGGTCTTACATGCACATC GCCACGGTGGGGTTTCTAAACTT | 900 900 | Heim et al 2003 |
| Enterovirus (5’ NTR) | Forward Reverse 1 Reverse 2 | TCCTCCGGCCCCTGA AATTGTCACCATAAGCAGCCA GATTGTCACCATAAGCAGCCA | 900 300 300 | Nijhuis et al 2002 |
| Human Metapneumovirus (Nuceloprotein) | Forward Reverse | CATATAAGCATGCTATATTAAAAGAGTCTC CCTATTTCTGCAGCATATTTGTAATCAG | 500 250 | Maertzdorf et al 2004 |
| Rhinovirus (5’NTR) | Forward 1 Forward 2 Reverse | CYAGCCTGCGTGGC TCAGCCTGCGTGGC GAAACACGGACACCCAAAGTA | 1000 1000 1000 | Modified from Lu et al 2008 |
| Parechovirus (5’UTR) | Forward 1 Forward 2 Reverse 1 Reverse 2 | GTAACAGRTGCCTCTGGGGCCAAAAG GTAACACYAGCCTCTGGGCCCAAAAG GGCCCCWGATCAGATCCAYAGT GGCCCCTGGTCAGATCCACAGT | 400 400 400 400 | Nix et al 2008 |
| Coronavirus NL63 (Nuceloprotein) | Forward Reverse | AGGACCTTAAATTCAGACAACGTTCT GATTACGTTTGCGATTACCAAGACT | 800 800 | Fouchier et al 2004 |
| Coronavirus HKU (Replicase 1b) | Forward Reverse | CCTTGCGAATGAATGTGCW TTGCATCACCACTGCTAGTACCAC | 600 650 | Dare et al 2007 |
| Corornavirus 229e (Nucleoprotein) | Forward Reverse | CAGTCAAATGGGCTGATGCA AAAGGGCTATAAAGAGAATAAGGTATTCT | 150 150 | Van der Elden 2004 |
| Coronavirus OC43 (Nuceloprotein) | Forward Reverse | CGATGAGGCTATTCCGACTAGGT CCTTCCTGAGCCTTCAATATAGTAACC | 450 450 | Van der Elden 2004 |
| K-RAS (Human Control) | Forward Reverse | GCCTGCTGAAAATGACTGAATATAAAC TGATTCTGAATTAGCTGTATCGTCAAG | 600 600 | Bibby et al 2011 |

**Supplementary Table 3. The function and resistance-conferring mechanism of the detected AMR genes in the resistome of the sequenced isolates, according to CARD database**

| **AMR Gene/ Mutation** | **Encoding protein/Function** | **Mechanism of resistance** | **Class of Antimicrobials affected** |
| --- | --- | --- | --- |
| *H. influenzae* | | | |
| *tem-1* | TEM-1: broad-spectrum β-lactamase | antibiotic inactivation | penicillins and first generation cephalosphorins |
| PBP3 - D350N | variant of penicillin binding protein 3 (PBP3) | antibiotic target alteration, | cephalosporins, cephamycins and penams |
| PBP3 - S357N | variant of penicillin binding protein 3 (PBP3) | antibiotic target alteration: | cephalosporins, cephamycins and penams |
| *lpsA* | LpsA: intrinsic peptide antibiotic resistant Lps which plays a role in lipopolysaccharide biosynthesis | reduced permeability to antibiotic | peptide antibiotics |
| *P. aeruginosa* | | | |
| *bla_OXA_* | OXA (class D β-lactamases) | antibiotic inactivation | β-lactam |
| *bla_PDC_* | PDC (class C β-lactamases) | antibiotic inactivation | β-lactam |
| *bla_PAO_* | class D β-lactamases | antibiotic inactivation | β-lactam |
| *aph(3')-IIb* | APH(3’)-11b: chromosomal-encoded aminoglycoside phosphotransferase | antibiotic inactivation | Aminoglycoside |
| *catB7* | chloramphenicol acetyltransferase | antibiotic inactivation | Phenicol |
| *crpP* | Ciprofloxacin phosphotransferase | antibiotic inactivation | fluoroquinolone (Ciprofloxacin) |
| *parE*_A473V* | fluoroquinolone resistant variant of ParE subunit of DNA topoisomerase IV | antibiotic target alteration | fluoroquinolone |
| *fosA* | fosfomycin, thiol transferase | antibiotic inactivation | fosfomycin, |
| *arnA* | PmrL: modifies lipid A with 4-amino-4-deoxy-L-arabinose (Ara4N) | antibiotic target alteration | cationic antimicrobial peptides (e.g. colistin and polymyxin |
| *cprRS* | A two-component regulatory system that induces *arn* operon in the presence of cationic peptides | antibiotic target alteration | cationic antimicrobial peptides (e.g. colistin and polymyxin |
| basR_ L71R* | Pmr: phosphoethanolamine transferase subunit | antibiotic target alteration, antibiotic efflux | peptide antibiotic |
| *basS/R* | BasRS (PmrAB*)* is a two-component regulatory system for *pmrE, pmrF and pmrC* | antibiotic target alteration, antibiotic efflux | peptide antibiotic |
| *amrR* | AmrR: antirepressor protein of MexR | upregulate MexAB-OprM. efflux system | multiple |
| MexS - S60P* | Variant that causes protein overexpression | antibiotic efflux | fluoroquinolone, diaminopyrimidine, phenicol |
| nalC - S209R, G71E* | Variant that causes  protein overexpression | antibiotic efflux | macrolide, fluoroquinolone antibiotic, β-lactam, tetracycline, diaminopyrimidine, sulfonamide, phenicol and peptide antibiotic |
| Type A NfxB | mutant of NfxB (MDR antibiotic efflux) that are 4–8x more resistant | antibiotic efflux | ofloxacin, erythromycin, and new zwitterionic cephems |
| Type B NfxB | mutant of NfxB (MDR antibiotic efflux) that are more resistant | antibiotic efflux | Tetracycline, chloramphenicol, ofloxacin, erythromycin, and new zwitterionic cephems |
| Gram Positive | | | |
| *ermB* | Erm 23S ribosomal RNA methyltransferase | antibiotic target alteration | macrolide, lincosamide, streptogramin |
| *msrD* | Mel: ATP binding cassette (ABC) homologous of MsrA | antibiotic target protection | macrolide, streptogramin |
| *mefA* | Major facilitator superfamily (MFS) antibiotic efflux pump | antibiotic efflux | macrolide, streptogramin |
| *tet(M)* | tetracycline-resistant ribosomal protection protein | antibiotic target protection | Tetracycline: doxycycline, tetracycline, minocycline |
| *tet (O)* | tetracycline-resistant ribosomal protection protein | antibiotic target protection | tetracycline |
| *tet (A/B)* | TetAB a heterodimeric ABC transporter | antibiotic efflux | tetracycline |
| *catQ* | chloramphenicol acetyltransferase (CAT) | antibiotic inactivation | phenicol antibiotic |
| *lsa(C)* | ABC-F ABC ribosomal protection protein | antibiotic target protection | macrolide, lincosamide, streptogramin, tetracycline, oxazolidinone, phenicol antibiotic |
| *pat(A/B)* | PatAB: ABC heterodimeric efflux protein complex | antibiotic efflux | Fluoroquinolone |
| *pmrA* | MFS antibiotic efflux pump | antibiotic efflux | Fluoroquinolone |
| *parC*_S82Y* | protein variant of ParC | antibiotic target alteration | Fluoroquinolone |

*Mutation MDR: Multi-drug Resistance

**References for Supplementary Tables**

- Chung WO, Werckenthin C, Schwarz S, Roberts MC. Host range of the ermF rRNA methylase gene in bacteria of human and animal origin. Journal of Antimicrobial Chemotherapy. 1999;43(1):5-14.
- Lina G, Quaglia A, Reverdy M-E, Leclercq R, Vandenesch F, Etienne J. Distribution of Genes Encoding Resistance to Macrolides, Lincosamides, and Streptogramins among Staphylococci. Antimicrobial Agents and Chemotherapy. 1999;43(5):1062-6.
- Luna VA, Cousin S, Jr., Whittington WL, Roberts MC. Identification of the conjugative mef gene in clinical Acinetobacter junii and Neisseria gonorrhoeae isolates. Antimicrob Agents Chemother. 2000;44(9):2503-6.
- Heim A, Ebnet C, Harste G, Pring‐Akerblom P. 2003. Rapid and quantitative detection of human adenovirus DNA by real‐time PCR. J Med Virol 70: 228–239.
- Maertzdorf J, Wang CK, Brown JB, Quinto JD, Chu M, de GM, van den Hoogen BG, Spaete R, Osterhaus AD, Fouchier RA. 2004. Real‐time reverse transcriptase PCR assay for detection of human metapneumoviruses from all known genetic lineages. J Clin Microbiol 42: 981–986.
- Bibby DF, McElarney I, Breuer J, Clark DA. Comparative evaluation of the Seegene Seeplex RV15 and real-time PCR for respiratory virus detection. J Med Virol. 2011;83(8):1469-1475.
- Van Elden L. J., Nijhuis M., Schipper P., Schuurman R., and van Loon A. M.. 2001. Simultaneous detection of influenza viruses A and B using real-time quantitative PCR. J. Clin. Microbiol. 39:196–200.
- Van de Pol AC, van Loon AM, Wolfs TF, Jansen NJ, Nijhuis M, Breteler EK, Schuurman R, Rossen JW. Increased detection of respiratory syncytial virus, influenza viruses, parainfluenza viruses, and adenoviruses with real-time PCR in samples from patients with respiratory symptoms. J Clin Microbiol. 2007 Jul;45(7):2260-2.
- Lu X, Holloway B, Dare RK, Kuypers J, Yagi S, Williams JV, Hall CB, Erdman DD. Real-time reverse transcription-PCR assay for comprehensive detection of human rhinoviruses. J Clin Microbiol. 2008 Feb;46(2):533-9.
- Kuypers J, Wright N, Morrow R. Evaluation of quantitative and type-specific real-time RT-PCR assays for detection of respiratory syncytial virus in respiratory specimens from children. J Clin Virol. 2004 Oct;31(2):123-9.
- Nijhuis M, van Maarseveen N, Schuurman R, et al. Rapid and sensitive routine detection of all members of the genus enterovirus in different clinical specimens by real-time PCR. J Clin Microbiol. 2002;40(10):3666-3670.
- Nix WA, Maher K, Johansson ES, Niklasson B, Lindberg AM, Pallansch MA, Oberste MS. Detection of all known parechoviruses by real-time PCR. J Clin Microbiol. 2008 Aug;46(8):2519-24.
- Ron A. M. Fouchier, Nico G. Hartwig, Theo M. Bestebroer, Berend Niemeyer, Jan C. de Jong, James H. Simon, and Albert D. M. E. Osterhaus. A previously undescribed coronavirus associated with respiratory disease in humans. PNAS.2004;April;101(16)6212-6216
- Dare RK, Fry AM, Chittaganpitch M, Sawanpanyalert P, Olsen SJ, Erdman DD. Human coronavirus infections in rural Thailand: a comprehensive study using real-time reverse-transcription polymerase chain reaction assays. J Infect Dis. 2007 Nov 1;196(9):1321-8.
